# Zinc-Modified Nanotransporter of Doxorubicin for Targeted Prostate Cancer Delivery

**DOI:** 10.3390/nano7120435

**Published:** 2017-12-08

**Authors:** Sylvie Skalickova, Martin Loffelmann, Michael Gargulak, Marta Kepinska, Michaela Docekalova, Dagmar Uhlirova, Martina Stankova, Carlos Fernandez, Halina Milnerowicz, Branislav Ruttkay-Nedecky, Rene Kizek

**Affiliations:** 1 Department of Human Pharmacology and Toxicology, Faculty of Pharmacy, University of Veterinary and Pharmaceutical Sciences Brno, 61200 Brno, Czech Republic; sylvie.skalickova@gmail.com; 2 Central Laboratory, Faculty of Pharmacy, University of Veterinary and Pharmaceutical Sciences Brno, 61200 Brno, Czech Republic; loffel.martin@gmail.com (M.L.); michaelgargulak@seznam.cz (M.G.); Michaeladocekalova@seznam.cz (M.D.); dagmar.uhlirova@seznam.cz (D.U.); MartStan@seznam.cz (M.S.); brano.ruttkay@seznam.cz (B.R.-N.); 3 Faculty of Pharmacy, Department of Biomedical and Environmental Analyses, Wroclaw Medical University, 50-556 Wrocław, Poland; zalewska.m@gmail.com (M.K.); halina.milnerowicz@umed.wroc.pl (H.M.); 4 Prevention Medicals s.r.o, Tovární 342, Butovice, 742-13 Studentka, Czech Republic; 5 School of Pharmacy and Life Sciences, Robert Gordon University, Garthdee Road, Aberdeen AB10 7QB, UK; c.fernandez@rgu.ac.uk

**Keywords:** doxorubicin, chitosan, zinc, sarcosine, ninhydrin, prostate cancer, peroxidase activity, gold nanoparticle, magnetic gold nanoparticle

## Abstract

This work investigated the preparation of chitosan nanoparticles used as carriers for doxorubicin for targeted cancer delivery. Prepared nanocarriers were stabilized and functionalized via zinc ions incorporated into the chitosan nanoparticle backbone. We took the advantage of high expression of sarcosine in the prostate cancer cells. The prostate cancer targeting was mediated by the AntiSar antibodies decorated surface of the nanocage. Formation of the chitosan nanoparticles was determined using a ninhydrin assay and differential pulse voltammetry. Obtained results showed the strong effect of tripolyphosphine on the nanoparticle formation. The zinc ions affected strong chitosan backbone coiling both in inner and outer chitosan nanoparticle structure. Zinc electrochemical signal depended on the level of the complex formation and the potential shift from −960 to −950 mV. Formed complex is suitable for doxorubicin delivery. It was observed the 20% entrapment efficiency of doxorubicin and strong dependence of drug release after 120 min in the blood environment. The functionality of the designed nanotransporter was proven. The purposed determination showed linear dependence in the concentration range of Anti-sarcosine IgG labeled gold nanoparticles from 0 to 1000 µg/mL and the regression equation was found to be *y* = 3.8*x* − 66.7 and R^2^ = 0.99. Performed ELISA confirmed the ability of Anti-sarcosine IgG labeled chitosan nanoparticles with loaded doxorubicin to bind to the sarcosine molecule. Observed hemolytic activity of the nanotransporter was 40%. Inhibition activity of our proposed nanotransporter was evaluated to be 0% on the experimental model of *S. cerevisiae*. Anti-sarcosine IgG labeled chitosan nanoparticles, with loaded doxorubicin stabilized by Zn ions, are a perspective type of nanocarrier for targeted drug therapy managed by specific interaction with sarcosine and metallothionein for prostate cancer.

## 1. Introduction

The use of biodegradable polymeric nanoparticles (NPs) for controlled drug delivery has shown significant therapeutic potential in several cancer diseases. One of the most utilized biopolymer is chitosan (CS) due to its beneficial chemical-physical properties [1]. Previous studies reported its antioxidant [2], anti-hypertensive [3], anti-diabetic, anti-obesity [4], neuroprotective [5] and bacteriostatic effects [6]. Moreover, chitosan plays an important role in the cell regulation and tissue regeneration [7,8]. From the chemical point of view, chitosan is a weak base polysaccharide with high amino-group density per disaccharide unit. In the dependence on pH of environment the amino groups ensure diverse molecular behavior. Generally, acidic pH causes protonation of amino-groups and positive charge. Simultaneously, the chitosan solubility increases when decreasing the value of pH [9]. 

The ability of chitosan gelation and crosslinking has been observed with several compounds. Protocols that employed aldehydes (e.g., glutaraldehyde) and acids (e.g., thioglycolic, acrylic and oxalic acid) can be found in the literature [10]. Ionic cross-links lead to nanoparticle formation which could be reached by electrostatic interaction with phosphoric acid derivatives such as sodium tripolyphosphate penta basic (TPP) which is a nontoxic, anionic chelating agent [11]. Chitosan’s ability of quick gelling on contact with TPP relies on the formation of inter- and intramolecular cross-linkages between TPP phosphates and chitosan amino groups. It was found out that the size and size distribution of the chitosan–TPP nanoparticles depends on concentration, molecular weight and conditions of mixing, i.e., stirring or sonication [12]. Gan et al. focused on the influence of TPP ratio: CS and pH on nanoparticle size and ξ-potential. The main results show the higher CS concentration and higher pH caused bigger particle size (in the range 140–180 nm based on the CS molecular weight) and increasing trend of ξ-potential from 30 to 50 mV. Authors explained this phenomenon by particle aggregation, rather than by further growth of the individual particle size after initial formation [13]. Complex information of CS–TPP interactions could provide circular dichroism spectroscopy [14], infra-red spectroscopy [15] or SEM/TEM techniques for size and morphology [16]. Colorimetric methods allow the study of chitosan interactions indirectly—e.g., ninhydrin assay, Cibacron Brilliant Red [17] or one-step depolymerization with sodium nitrite followed by the reaction of the end product with thiobarbituric acid [18].

The positive charge of CS NPs enables to bind negative charged biomolecules via electrostatic interaction. These properties have been used in several studies focused on several delivery systems including drugs, genes or nanoparticle delivery to the target tissues which is well described in many reviews [19,20,21,22,23,24]. Lee et al. demonstrated the chemical conjugation of CD^7−^ specific single-chain antibody on the CS nanoparticle backbone useful for the delivery of siRNA into CD^4+^ T cell lines [25]. Generally, monoclonal antibodies are clinically established therapeutics and presents perspective strategy emerging to target them for more effective treatment of cancer [26]. Tumor tissue is characterized by different metabolism, higher expression of specific biomolecules and changed morphology. In 2009 Sreekumar published a crucial article about potential role of sarcosine in the prostate cancer. Metabolomic profiling of prostate cancer (PC) progression identified markedly elevated levels of sarcosine (*N*-methyl glycine) in metastatic PC and modest but significant elevation of the metabolite in tissue and urine [27]. Moreover, sarcosine higher levels have been confirmed in thyroid [28] and breast cancer [29]. 

Many studies also indicate higher expression of metallothionein (MT) in the case of prostate cancer [30]. MT belongs to a family of low molecular weight proteins that are involved in zinc and redox metabolism as well as in many aspects of cancer biology and resistance to many toxic drugs [31]. In our previous published work, we have shown significant increases of MT in malignant tumors [32]. Moreover, the higher zinc uptake by tumor tissue has been noted [33]. 

**Figure 1:**
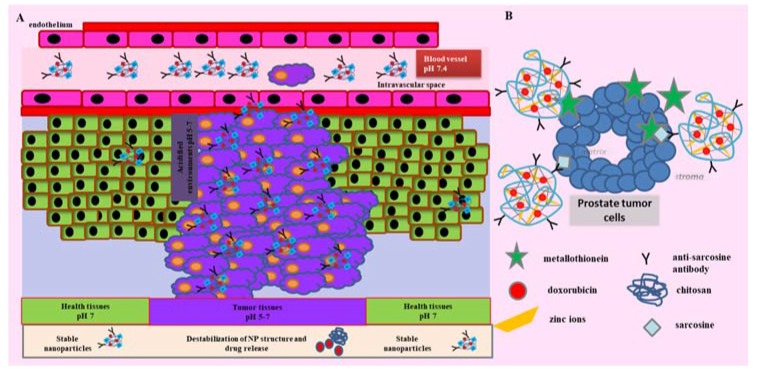
(**A**) Schematic diagram of the effect of chitosan nanoparticles for the complex of zinc, doxorubicin and specific antibodies. Tumor tissue is acidic environment (pH 5–7), which leads to destabilization of the NP structure and drug release. Chitosan nanoparticles are modified by an antibody specific to tumor-associated molecules. In a healthy tissue, a nanotransporter penetrates but the destabilization of the nanotransporter is limited. Healthy cells do not express the tumor antigen and metallothionein, the protein of the molecule and thus there is no targeted binding on the cell surface. (**B**) Scheme of CS-Zn-AntiSar nanoparticles formation for doxorubicin delivery to prostate tumor cells.

Although, the progression of the cancer disease therapy has been given, the importance of finding the effective cancer treatment approach is still the main field of many researchers. The aim of our study was to design a chitosan based nanotransporter for anticancer drugs doxorubicin. We assumed the positive effect of zinc ions in the nanoparticle structure for its stability, target effect to MT, specific interaction with AntiSar antibody and increased uptake of the nanotransporter from the bloodstream (Figure 1). Direct targeting for prostate cancer ensures the presence of AntiSar antibodies. Function and stability of our designed nanotransporter was investigated by the spectrophotometric and electrochemical approaches.

## 2. Results

### 2.1. Biophysical Characterization of CS Nanoparticles

**Figure 2:**
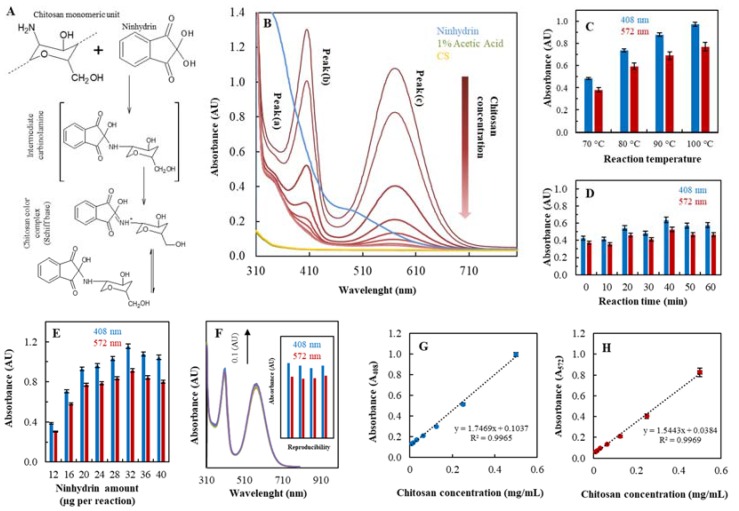
Biophysical characterization of CS NPs. (**A**) The principle of ninhydrin reaction with chitosan. (**B**) Absorbance spectra of ninhydrin-CS complex (CS concentration 0–0.5 mg/mL), ninhydrin reagent and 1% acetic acid. Optimization of the method: (**C**) reaction temperature (70, 80, 90, 100 °C). (**D**) reaction time (0, 10, 20, 30, 40, 50, 60 min) (**E**) ninhydrin amount per reaction (12, 16, 20, 24, 28, 32, 36, 40 µg). (**F**) Reproducibility of assay presented as absorbance spectra and the slope of each value in A_408_ nm and A_572_ nm (*n* = 5). Dependence of CS concentration (0–0.6 mg/mL) on absorbance (**G**) A_408_ nm. (**H**) A_573_ nm, 3 times repeated. The measurement conditions for all samples were: ninhydrin amount per reaction 32 µg, CS concentration 0.25 mg/mL, reaction time 40 min and reaction temperature 100 °C unless noted otherwise.

For characterization of the formed nanoparticles and the influence of zinc ions and TPP on nanoparticles formation spectrophotometric analysis and electrochemistry have been used. While electrochemistry provide data about zinc availability in the nanoparticle structure, the spectrophotometric analysis is based on the reaction of CS with ninhydrin solution which is enabled to react with primary and secondary amino groups. The chromophore is created by amine condensation with a molecule of ninhydrin to give Ruhemann purple (Figure 2A). From absorbance spectra (Figure 2B) of ninhydrin-CS complex three characteristic absorbance maxima in (a) 342, (b) 408 and (c) 572 nm are obvious. While absorbance maximum A_408_ and A_572_ corresponds to the Rhueman purple complex the linear relationship with the CS concentration has been observed. The absorbance peak at 342 nm appears when the CS-ninhydrin reaction is stopped by aqueous ethanol solution [34]. 

#### 2.1.1. Ninhydrin Assay Optimization

Firstly, the effects of several factors including the reaction temperature, reaction time and the ninhydrin concentration on the chitosan-ninhydrin reaction were investigated to optimize the specificity and sensitivity. Figure 2C shows the dependence of reaction temperature 70, 80, 90 and 100 °C where is obvious the increase of chromophore absorbance in correlation with higher temperature in both absorbance maximum A_408_ and A_572_. The reaction temperature was set on 100 °C for testing the reaction time (0, 10, 20, 30, 40, 50, 60 min). The results (Figure 2D) clearly shown the reaction time up to 40 min leads to the highest absorbance which is stable after 50 and 60 min at A_408_ and A_572_. The dependence of ninhydrin amount entering the reaction with chitosan was investigated. Figure 2E shows an increasing trend of signal in correlation with the increasing ninhydrin amount up to 32 µg per reaction. Higher concentration caused slight decrease of the signal both in A_408_ and A_572_. We chose the optimal conditions of ninhydrin assay for chitosan detection (100 °C reaction temperature, 40 min reaction time and 32 µg on ninhydrin per reaction) to test ninhydrin assay reproducibility. In the Figure 2F absorbance spectra present 4 times repetition of the analysis and added inset illustrates each measurement of absorbance maximum at A_408_ and A_572_. The calculated relative standard deviation is 2% (Table 1). The dependence of chitosan concentration (0–0.5 mg/mL) on absorbance A_408_ and A_572_ is shown in Figure 2G,H respectively. Calculated limit of detection was 2 µg/mL for A_408_ and A_572_. Other analytical parameters such as LOD, LOQ, Linear range are summarized in Table 1.

#### 2.1.2. Characterization of Zn-TPP-CS Nanoparticles Using Ninhydrin Assay

**Figure 3:**
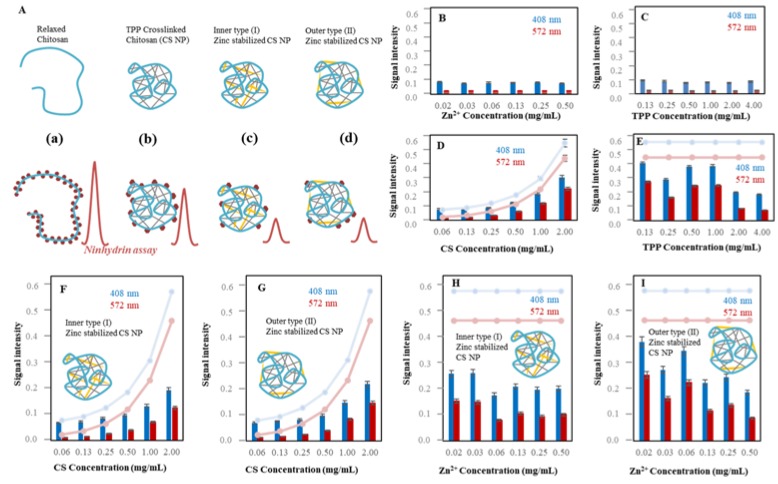
Spectrophotometrical characterization of CS NPs. (**A**) Characterization of Zn-TPP-CS nanoparticles using ninhydrin assay. The relaxed CS has accessible NH_2_ groups for ninhydrin reaction. TPP and Zn caused crosslinking affecting the structure stabilization and decreasing signal intensity. Two types of NPs have been designed: Zn inner type (I) and Zn outer type (II). The effect of changing concentration of each component of NPs has been observed using the ninhydrin assay. The signal of CS itself is interposed to the sample absorbance (light blue and red lines): (**B**) zinc (0.02, 0.03, 0.06, 0.13, 0.25, 0.50 mg/mL), (**C**) TPP (0.13, 0.25, 0.50, 1.00 2.00, 4.00 mg/mL), (**D**) CS (0.06, 0.13, 0.25, 0.50, 1.00, 2.00 mg/mL) and 2.00 mg/mL TPP, (**E**) TPP (0.13, 0.25, 0.50, 1.00, 2.00, 4.00 mg/mL) and 2.00 mg/mL CS. (**F**) Inner type (I): CS (0.06–2 mg/mL) with Zn (0.5 mg/mL), addition of TPP (2 mg/mL), (**G**) Outer type (II): CS (0.06, 0.13, 0.25, 0.50, 1.00, 2.00 mg/mL) with TPP (2 mg/mL), addition of Zn (0.50 mg/mL). (**H**) Inner type (I): Zn (0.02, 0.03, 0.06, 0.13, 0.25, 0.5 mg/mL) with CS (2 mg/mL), addition of TPP (2 mg/mL). (**I**) Outer type (I): TPP (2 mg/mL) with CS (2 mg/mL), addition of Zn (0.02, 0.03, 0.06, 0.13, 0.25, 0.50 mg/mL). Further details are described in Figure 2 and Materials and Methods section.

We used ninhydrin assay to monitor chitosan properties in the presence of TPP and zinc ions. As was described in the introduction, TPP as well as zinc ions stabilize chitosan crosslinked structure and provide nanoparticle formation. The scheme in Figure 3A demonstrates the principle of use ninhydrin assay to characterize CS nanoparticles. The relaxed structure of chitosan (a) has an accessible amino group for ninhydrin reaction. While the TPP is added (b) the structure is strongly crosslinked and the nanoparticles are formed. The number of accessible amino groups decreases proportionally with the level of crosslinking and the structure of formed nanoparticles. This behavior results to less accessible amino groups which cause the lower absorbance signal of ninhydrin [35,36]. Zinc ions could be added in two ways. Firstly, the zinc is added before TPP reaction with chitosan. In this case could be assumed, the zinc ions stabilize the inner structure (c) of chitosan NP whereas the zinc ions added after the TPP crosslinking probably stabilize the structure by outer backbone (d).

Firstly, we wanted to exclude interaction of zinc or TPP with ninhydrin. From the Figure 3B,C is obvious the equal signal intensity for all applied concentrations comparable to ninhydrin signal intensity in both A_408_ and A_572_. In order to see the influence of TPP and zinc on the crosslinking effect, the results are interleaved by the signal of chitosan alone (A_408_ light blue curve and A_572_ light red curve). The greater the difference in signal, the greater the crosslinking effect was estimated. Figure 3D is shows the dependence of CS concentrations from 0.06 to 2.00 mg/mL in the presence of 2 mg/mL TPP. The low CS concentration in the presence of TPP shows similar signal intensity as CS alone. From 0 to 0.5 mg/mL is evident that the signal intensity is lower than CS alone. The CS NPs are well formed in the concentration 2 mg/mL of CS and the higher TPP concentration caused its stabilization. From Figure 3E the apparent signal intensity of CS NPs decreases at higher TPP concentration that results to more crosslinked structure. Further results are focused on comparison of inner and outer type of NPs stabilized by zinc ions. In the case of Zn-inner type of NPs, zinc ions are inside the CS structure and in the case of outer type of NPs, the zinc on the NPs surface. Both types are the result of the different preparation protocol described in the materials and methods caption. Figure 3F (inner type) and Figure 3G (outer type) shows similar trend of signal intensity in the dependence of CS concentration. In this case we can assume that the applied CS concentration did not influence the stability of the NP. Interestingly, higher effect to NPs formation shows zinc ions addition. In both cases (Figure 3I), the zinc addition (from 0.02 to 0.5 mg/mL) caused signal intensity decrease in comparison with the CS itself, which indicates that the structure is more closed and stabilized. In addition, the zinc ions addition before TPP crosslinking shows higher efficiency of Zn-CS NPs stabilization (Figure 3H) which results in an overall lower signal of NPs compared to CS (2 mg/mL).

#### 2.1.3. Electrochemical Study of CS NPs Formation

**Figure 4:**
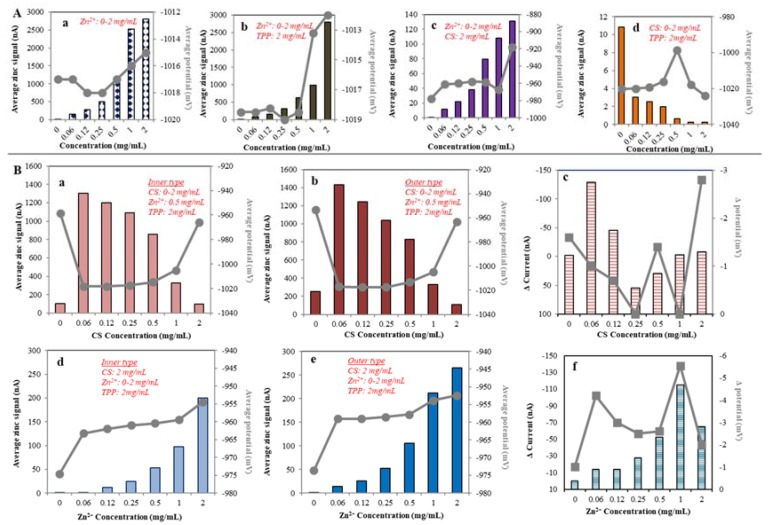
Electrochemical characterization of CS NPs. (**A**) Interaction study of CS-TPP-Zn complex. Analyses were carried out using difference pulse voltammetry. (**a**) Influence of zinc concentration (0.02, 0.03, 0.06, 0.13, 0.25, 0.50 mg/mL) on average zinc ions signal (peak s V). (**b**) Influence of CS concentration (0.06, 0.13, 0.25, 0.50, 1.00, 2.00 mg/mL) in the mixture with TPP (2 mg/mL). (**c**) Influence of increasing zinc ions concentration (0.06, 0.13, 0.25, 0.50, 1.00, 2.00 mg/mL) and 2 mg/mL TPP. (**d**) Influence of increasing zinc ions concentration and CS (2 mg/mL) on average zinc ions signal. (**B**) Influence of CS and zinc ions on the CS NPs formation and the (**a**) Inner type (I): CS (0.06, 0.13, 0.25, 0.50, 1.00, 2.00 mg/mL) with zinc ions (0.50 mg/mL), addition of TPP (2 mg/mL), (**b**) Outer type (II): CS (0.06, 0.13, 0.25, 0.50, 1.00, 2.00 mg/mL) with TPP (2 mg/mL), addition of zinc ions (0.50 mg/mL). (**c**) Inner type (I): zinc ions (0.02, 0.03, 0.06, 0.13, 0.25, 0.50 mg/mL) with CS (2 mg/mL), addition of TPP (2 mg/mL). (**d**) Outer type (I): TPP (2 mg/mL) with CS (2 mg/mL), addition of zinc ions (0.02, 0.03, 0.06, 0.13, 0.25, 0.50 mg/mL). Difference zinc ions signal for (**e**) Inner type NPs, (**f**) Outer type NPs. Further details are described in Figure 3 and Materials and Methods section.

The dependence of zinc concentration (from 0 to 2 mg/mL) on the electrochemical signal is shown in Figure 4A(a). In the case of zinc-TPP interaction, the potential shift is the same as well as zinc alone −1.017 V. However, the electrochemical response increases with the concentration of zinc ions. When the Zn^2+^ concentration increases from 0.06 mg/L to 1 mg/mL the electrochemical response decreases in two folds compared to Zn^2+^ alone (Figure 4A(b)). In another variant, to the 2 mg/mL CS was added zinc ions in the concentrations 0.02, 0.03, 0.06, 0.13, 0.25, 0.50 mg/mL). The graph Figure 4A(c) shows rapid decrease of zinc electrochemical signal. This effect could be caused by CS adsorption on the electrode surface. The difference is clearly shown in the zinc peak potential shift followed by increasing zinc concentration (Figure 4A(c)). Finally, the same method was used for the determination of CS and TPP interactions. Summarized results showed the weak electrochemical signal (in order of units of nA) and the influence of CS on decreasing the electrochemical signal (Figure 4A(d)).

We used electrochemistry to investigate the behavior of the CS nanotransporter and influence of zinc ions to stabilize its structure outside and inside the transporter. In the inner type of CS NP, the zinc was added before TPP crosslinking whereas the outer type is characterized by the presence of zinc ions on the nanoparticle surface due to the zinc addition after the TPP gelation process. Figure 4B(a) shows the influence of increasing CS concentration (0.06, 0.13, 0.25, 0.50, 1.00, 2.00 mg/mL) on zinc (0.50 mg/mL) signal which exhibit decreasing dependence in the case of Zn inner type of nanotransporter. Monitored peak potential was in the range from −960 to −950 mV, that means good agreement with the inner type of CS NPs. Although, the increased CS concentration correlated with the electrochemical signal in both inner and outer type of zinc stabilized nanotransporter, the higher electrochemical signal is observed in the case of outer type (Figure 4B(b)). These results confirmed the zinc stabilization function on the nanoparticle surface. More prominent differences can be seen after subtraction of both electrochemical signals. Figure 4B(b,c) shows an apparent difference between each type of the nanotransporter. The increasing CS concentration (0.06, 0.13, 0.25, 0.50, 1.00, 2.00 mg/mL) causes higher difference up to 0.12 mg/mL followed by strong difference decrease. In addition, the influence of zinc ions concentration (0.02, 0.03, 0.06, 0.13, 0.25, 0.50 mg/mL) was studied in the dependence of electrochemical behavior of CS NPs transporter. In both cases, the increasing of zinc concentration caused higher electrochemical signal of zinc ions and the peak potential shift. The outer type of zinc ions stabilized the nanotransporter, showing the higher electrochemical response (Figure 4B(b,d,f)). Data was expressed as a difference of the inner type and outer type nanotransporter electrochemical signal on the Figure 4B(f). The apparent increase of the electrochemical signal in the concentration of zinc ions ranges from 0.06 to 1.00 mg/mL. This range expresses the weaker influence of CS to decrease the electrochemical signal. At higher zinc concentrations, there is a reduction in the difference between inner and outer type of Zn/CS NPs due to the fact that nanoparticles are unable to bind more zinc into its structure. 

### 2.2. Doxorubicin Encapsulation to the CS NPs Structure

**Figure 5:**
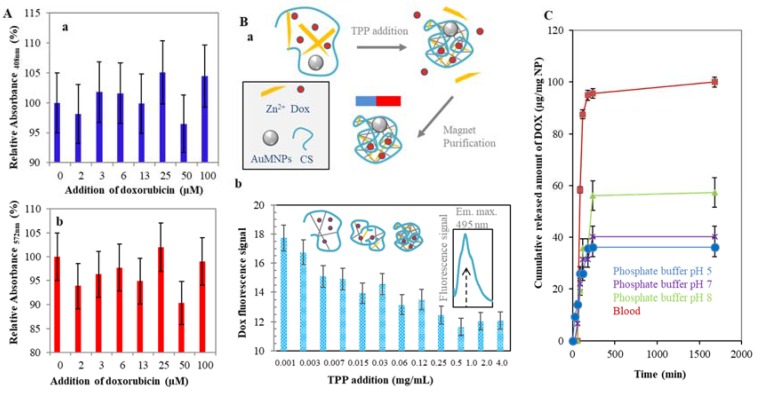
Doxorubicin encapsulation into Zn/CS NPs—biophysical characterization. (**A**) Encapsulated Dox (0, 2, 3, 6, 13, 25, 50, 100 µM) into the Zn/CS NPs structure and its effect on the ninhydrin assay. The signal at 408 nm (**a**) and 572 nm (**b**) have been compared. (**B**) Scheme of AuMNPs doped Zn/CS NPs preparation. To the mixture of CS, Zn^2+^ and Dox, AuMNPs were added and incubated. After TPP addition the CS NPs was formed and unbounded parts were washed using magnet purification (**a**). Effect of TPP addition (0.001, 0.003, 0.007, 0.015, 0.03, 0.06, 0.12, 0.25, 0.5, 1.0, 2.0 and 4.0 mg/mL) on doxorubicin fluorescence signal. The presence of AuMNPs enabled the purification of complex from unbounded parts. Inset if the fluorescence spectra of Dox with the emission maximum at 495 nm (**b**). (**C**) Dependence of cumulative release of Dox (3 µM) on the time (min) in various environment: 10 mM phosphate buffer pH 5 (●), pH 7 (*), pH 8 (▲) and whole blood (■). Further details are described in Figure 3 and Materials and Methods section.

The described CS nanotransporter is primarily designed for drug delivery to target cancer tissue. For this purpose, doxorubicin was tested as one of the most used medicines for cancer diseases. To eliminate the influence of the doxorubicin ninhydrin test—which is one of four basic approaches to the characterization of Zn/CS NPs—the effect of encapsulated doxorubicin on the spectrophotometric signal of Ruhemann’s purple was investigated. Graphs in Figure 5A show the dependence of doxorubicin addition (0, 2, 3, 6, 13, 25, 50, 100 µM) on the absorbance at 408 nm (a) and 572 nm (b) which is characteristic absorbance maxima of the Ruhemann’s purple. Given values are recalculated as 100 percent absorbance of CS-ninhydrin product without Dox. Obtained data of both signals show the variability from 5 to 10%. These results indicate the formed complex is stable and the doxorubicin has no effect on Zn/CS NPs stabilization. 

In order to monitor the drug release from the nanotransporter, the further manipulation with the nanotransporter to purify it from the unbound parts of the complex was mediated via gold magnetic nanoparticles (AuMNPs). AuMPs could easily be encapsulated inside the NPs cage before adding the crosslinking agent. A maghemite core gives the nanoparticles magnetic properties and the gold layer on the surface mediates interactions with amino groups of CS. A brief scheme of the AuMNPs enclosing the structure of Zn/CS NPs/Dox-AuMNPs is shown in the Figure 5B(a). Magnetic Zn/CS nanoparticles are immobilized via external magnet and the unbounded parts of the mixture could be washed. The described system was used for the encapsulation efficiency evaluation. TPP was added in various concentrations to the Zn/CS mixture. After the crosslinking process, the magnetic Zn/CS NPs/Dox was transferred to the fresh buffer (10 mM phosphate buffer, pH 5) and the fluorescence of the doxorubicin was measured. Figure 5B(b) illustrates a decreasing trend of the doxorubicin fluorescence with the higher concentration of TPP. This could be attributed to the stronger crosslinking of the nanoparticle structure. 

Doxorubicin carried by Zn/CS NPs can be released through degradation of polymeric surface, leading to a clear release effect. It has been described that drug release of CS based carriers depends on the external conditions such as temperature, ionic strange, matrix composition and pH of the environment [37]. Study of release mechanisms of nanoparticle cargo is one of the basic aspects of NPs characterization. The Zn/CS NPs stability and doxorubicin release at intervals of several hours to several days was investigated [38]. Zn/CS NPs were subjected for 28 h to doxorubicin fluorescence sensing and the cumulative released amount of Dox was calculated. Figure 5C presents the obtained dependence of time on cumulative doxorubicin release amount in selected environment conditions: blood, phosphate buffers (10 mM) pH 5, 7 and 8. Results clearly show the rapid Dox release began immediately and ends after 250 min. Further Dox release is not intensive and the NPs are stable. An obvious difference between each environment shows the highest stability of Zn/CS NPs in pH 5 and 7. The strongest cumulative released of Dox amount is observed in the blood environment. The binding affinity of CS/NPs on erythrocytes and the loss of nanotransporter during purification was observed. 

### 2.3. Labeling of CS NPs by AntiSar Antibodies

The scope was to design nanocarrier which can target into the cancer tissue with highly expressed levels of sarcosine, such as prostate cancer. Non-specific conjugation of AntiSar antibody to Zn/CS NPs surface was utilized. This interaction is mediated by electrostatic and hydrophobic interactions between positively charged CS and proteins [39]. The interaction verification was employed to the dot blot analysis as a rapid and precise immunochemical method. Polyclonal chicken AntiSar antibodies as an anchor for targeting Zn/CS NPs/Dox was utilized. Monoclonal rabbit AntiChicken antibodies labeled with gold nanoparticles (AuNPs) were used as secondary antibodies. Based on our previous studies the AuNPs was employed as a detection system due to its peroxidase activity and ability to oxidize TMB substrate accompanied by color reaction [40]. 

**Figure 6:**
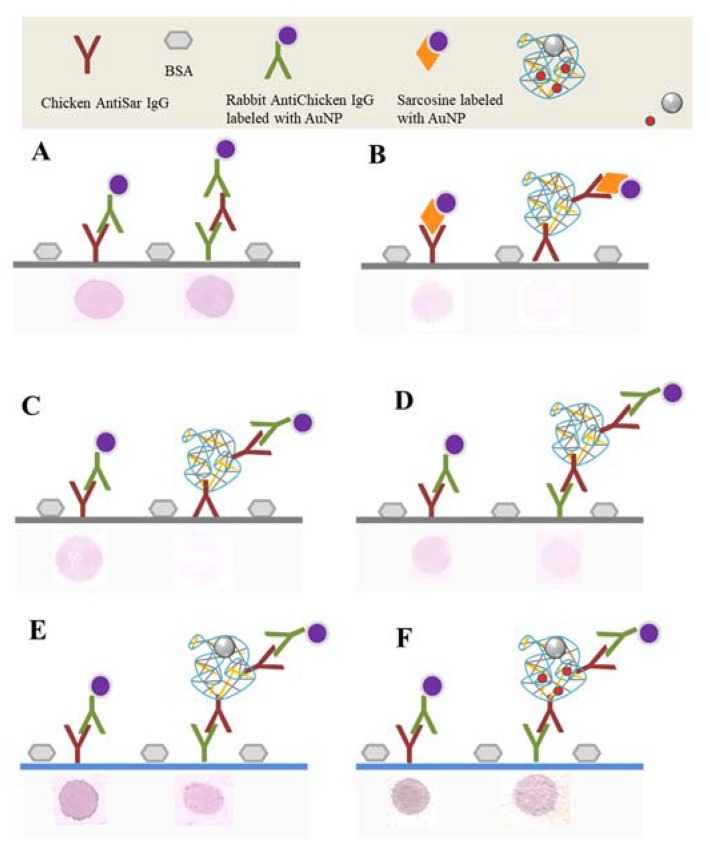
Dot blot assay of AntiSar/Zn/CS NPs. On the left side, all results from the control sample are presented. (**A**) Testing the functionality of Chicken AntiSar and Rabbit AntiChicken IgG. As a detection system, the AuNPs IgG was used. (**B**) Ability of CS NPs to bind Chicken AntiSar IgG. As a detection system, the AuNPs labeled sarcosine was used. (**C**) AntiSar/Zn/CS NPs detection via Rabbit AntiChicken IgG labeled with AuNP. Interaction of Rabbit AntiChicken IgG with (**D**) AntiSar/Zn/CS NPs, (**E**) AntiSar/Zn/CS NPs-AuMNPs and (**F**) AntiSar/Zn/CS NPs/Dox-AuMNPs detection via Rabbit AntiChicken IgG labeled with AuNP. The principle of detection was the aggregation of AuNPs after 20 min. All tested membranes were blocked by 1% BSA for 30 min in 22 °C and rotation 30 rpm. Between each experimental step membranes were washed 3-times in 5 mL PBS-T, pH 7) to purify the unbounded parts. Concentration of used AuNPs for sample labeling was 125 mg/mL, chicken antibodies 30 mg/mL and antichicken antibodies 40 mg/mL. All experiments were carried out in three replicates.

To confirm the binding of the AntiSar antibodies on the Zn/CS NPs surface the functionality of the Chicken AntiSar and Rabbit AntiChicken antibodies labeled with AuNPs using the dot blot analysis were tested. The ability of AuNPs aggregation which is visible as a purple dot on the membrane was utilized. After initiation of the primary antibodies, the membrane surface was blocked by a bovine serum albumin (BSA) to avoid false positive results. The dot blot analysis shows a positive signal in a both cases; the control (rabbit AntiChicken IgG labeled with AuNP) and the binding of both antibodies (chicken and rabbit antibodies) (Figure 6A). It could be assumed that the proposed system is applicable and therefore, it can be proceeded with chitosan testing itself. The chicken AntiSar antibodies were attached to the surface of Zn/CS NPs. The formation of this complex was confirmed by the interaction with AuNPs labeled sarcosine (AuSar). Figure 6B shows a positive signal of the control sample and a weakly colored dot of gold sarcosine, 200 µg/mL. Similar results were achieved if the selected detection system is rabbit or AntiChicken antibodies labeled by AuNPs (Figure 6C). The stronger interaction was occurred when primary rabbit AntiChicken antibodies were immobilized on the membrane. Next, we proceeded to test the sandwich method. The Zn/CS NPs-AntiSar labeled by rabbit AntiChicken-AuNPs showed the strong positive reaction (Figure 6D). 

To quantify the detection signal, the method from the membrane to the microplate was transferred which enabled the spectrophotometric detection. For this experimental purpose, magnetic Zn/CS NPs were employed. This system allows us to purify the sample from unbounded parts of the complex. Figure 6E,F show strong positive signal of Rabbit AntiChicken-AuNPs in the empty nanocarrier and with Dox. The results confirm the functionality of the bound antibodies to the Zn/CS NPs in vitro.

**Figure 7:**
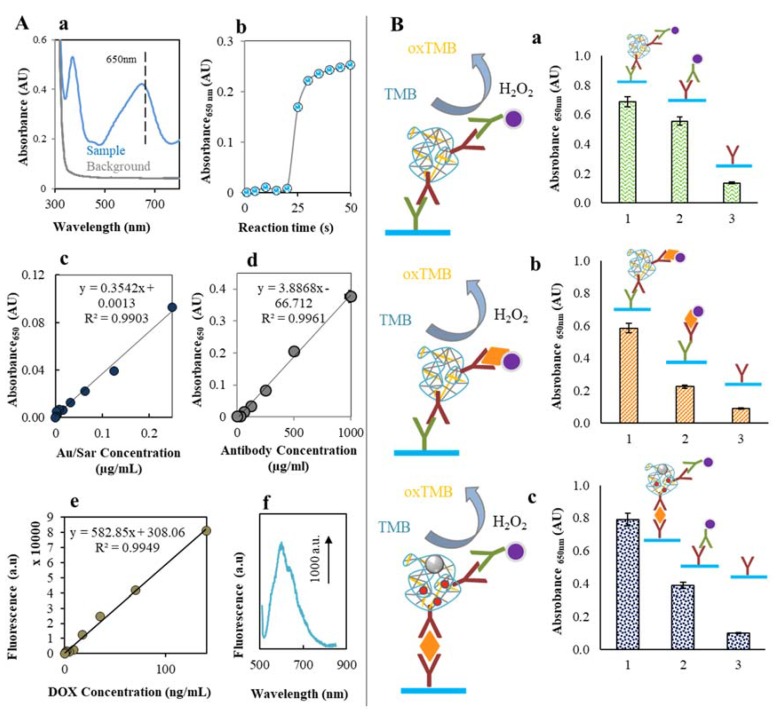
(**A**) Spectrophotometric characterization of AuNPs-AntiSar IgG pseudoperoxidase activity. (**a**) Characteristic spectra of AuNPs peroxidase activity in the range of 300–800 nm. (**b**) Typical reaction curve of AntiSar/Zn/CS AuNPs nanoparticles. Amount of detected antibody in the complex was in the range 20–40%, the signal of CS was subtracted. (**c**) Dependence of AuSar concentration (0, 0.002, 0.004, 0.008, 0.016, 0.03, 0.06, 0.1 and 0.25 mg/mL) on absorbance at 650 nm. (**d**) Dependence of signal intensity at A_650_ on the chicken AntiSar antibody concentration (0, 1, 2, 4, 8, 16, 31, 66, 125, 250, 500 and 1000 µg/mL). (**e**) Dependence of signal intensity of Dox encapsulated into the AntiSar/Zn/CS NPs nanotransporter (0, 1, 2, 4, 9, 18, 35, 70 and 140 ng/mL) and (**f**) characteristic doxorubicin fluorescence spectra. Amount of bonded Dox was among 5–10%. (**B**) ELISA methodology of AntiSar/Zn/CS NPs in various arrangements. (**a**) Left: scheme of experiment. AntiSar/CS NPs (complex) was bonded with rabbit AntiChicken IgG (1 mg/mL) attached on polystyrene microplate. The detection was carried out using pseudoperoxidase activity of Au-NPs/AntiChicken IgG. Right: peroxidase activity of each tested samples: (1) control sample (2), bonded AntiSar antibody (3) complex. (**b**) Left: scheme of experiment. AntiSar/Zn/CS NPs (complex) was bonded with rabbit AntiChicken IgG (1 mg/mL) attached on polystyrene microplate. The detection was carried out using pseudoperoxidase activity of Au-NPs/sarcosine. Left: peroxidase activity of each tested samples: (1) control sample (2), bonded AntiSar antibody (3) complex. (**c**) Left: scheme of experiment. AntiSar/Zn/CS NPs/Dox-AuMNPs (complex) was bonded to sarcosine (0–1 mg/mL) attached to rabbit AntiChicken IgG (1 mg/mL) immobilized on polystyrene microplate. The detection was carried out using pseudoperoxidase activity of Au-NPs/AntiChicken IgG. Right: peroxidase activity of each tested samples: (1) control sample (2), bonded AntiSar antibody (3) complex. The reaction conditions were as follows: 0.05 mM TMB, 0.1 mM acetate buffer pH 4 and 0.03% H_2_O_2_.

In further experiment, we used ELISA system to study the interaction of AntiSar antibodies and Zn/CS NPs. AuNPs show pseudoperoxidase activity, which leads to the oxidation of the TMB substrate. Figure 7A illustrates the photometric detection of the AntiSar/Zn/CS NPs. The characteristic absorbance spectrum of the ox/red TMB reaction Sar/Au is illustrated in Figure 7A(a). After 45 min two absorbance maxima peaks at 375 and 650 nm could be observed. The absorbance maximum at 650 nm belongs to the product of the enzymatic reaction between AuNPs and TMB in the presence of H_2_O_2_. Characteristic reaction time-dependent course is showed in Figure 7A(b). From the reaction curve is obvious a rapid increase of absorbance (*λ* = 650 nm) after 20 s. From 30th second the slight increase of the absorbance occurred and the reaction slowly goes down. 

Each component of the reaction system has been analyzed. The dependence of gold nanoparticles labeled sarcosine (Sar/Au) on the absorbance shows linear trend in the range from 0.9 to 250 ng/mL with the R^2^ = 0.99 and regression equation *y* = 0.3542*x* + 0.0013 (Figure 7A(c)). Further to Au/Sar analysis the peroxidase activity of the Sar/Au attached to Anti/Sar antibodies was also monitored. The dependence of labeled Anti/Sar antibodies concentration on the absorbance showed a linear trend over the range from 7.8 to 1000 µg/mL. The regression equation was found to be *y* = 3.8868*x* − 66.712 and R^2^ = 0.99 (Figure 7A(d)). The calculated concentration of Anti/Sar antibody was found to be between 20 and 40% in the analyzed complex (chitosan signal was subtracted). 

AuMNPs were used as a tool to immobilize the nanotransporter complex via magnet which enable the purification of the NPs from the unbounded parts. The dependence of encapsulated doxorubicin concentration on the fluorescence signal is shown in Figure 7A(e). The determined amount of encapsulated Dox in the Zn/CS NPs structure is in the range from 5 to 10%. Figure 7A(f) shows the characteristic fluorescence spectra of Dox in the presence of Zn/CS NPs and AuMNPs. 

Further, formation of the Zn/CS NP-antibody complex in ELISA system was proved. Two detection schemes were designed for this purpose (a) using AuNPs labeled Anti/Sar Antibodies and (b) using AuNPs labeled sarcosine (Sar/Au). After the TMB addition, the peroxidase activity was evaluated in both cases (a,b) in the three parts of complex: 1—background presented as a Chicken AntiSar antibodies, 2—control sample composed of AuNPs labeled sarcosine or AuNPs labeled AntiSar antibodies and 3—whole complex. As is shown in Figure 7B(a,b), the highest signal occurs in the case of formed complex Zn/CS NPs-AntiSar antibodies for both detection systems, in comparison with the background. The control sample shows positive signal in both cases this could be attributed to the functionality of the detection system. 

Finally, the binding ability of Zn/CS NPs with sarcosine was evaluated. On the polystyrene surface, the Chicken AntiSar antibodies were immobilized and in the presence of sarcosine (1 mg/mL) the Zn/CS NPs/AntiSar formed a sandwich as is demonstrated in the scheme in Figure 7B(b,c). Low peroxidase activity is estimated in the case of background sample (bonded AntiSar antibodies). Higher peroxidase activity signal of control sample signalized the presence of AuNPs labeled AntiSar antibodies. Whole complex shows the highest peroxidase activity and confirm the function of Zn/CS NPs/AntiSar nanoparticles to bind sarcosine. From these unique results it could be concluded, that our designed chitosan nanocarrier for doxorubicin (or other anthracycline antibiotic) is able to bind sarcosine which is highly expressed in the prostate cancer tissue.

### 2.4. Toxicity Evaluation of CS NPs

**Figure 8:**
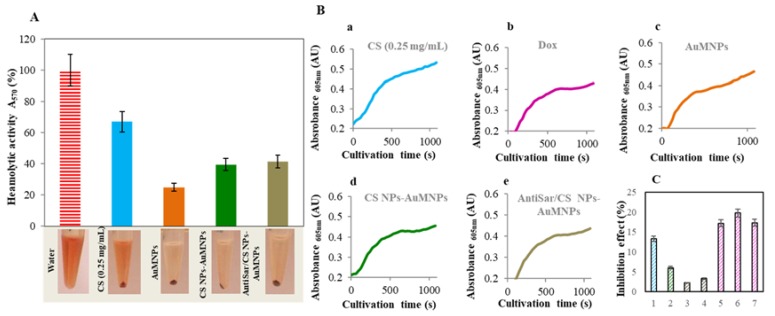
Study of toxicity Zn/CS NPs/Dox-AuMNPs. (**A**) Determination of hemolytic activity of prepared nanoparticles. The number of erythrocytes was 2.42 × 10^12^. Tested variants: water, CS (0.25 mg/mL), AuMNPs (1 mg/mL), Zn/CS NPs/Dox-AuMNPs (Dox = 6 µM) and AntiSar/Zn/CS NPs/Dox-AuMNPs (Dox = 6 µM). Inset photographs of each variant (centrifugation 2000 rpm, 5 min. to a completely clear solution). The pellet was diluted by PBS. The supernatant was spectrophotometrically analyzed, the absorbance spectra was obtained and the results were evaluated in 570 nm. Experiment was carried out using two replicates. (**B**) Toxicity evaluation of complexes using eukaryotic cells *Saccharomyces cerevisiae*. Characteristic growth curves for CS (0.25 mg/mL), AuMNPs (1 mg/mL), Zn/CS NPs/Dox-AuMNPs, AntiSar/Zn/CS NPs/Dox-AuMNPs and Dox (0.75 µM). (**C**) Average inhibition effect of samples: (0) *S. cerevisiae*, (1) AuMNPs (1 mg/mL), (2) CS (2 mg/mL), (3) Zn/CS NPs/Dox-AuMNPs (Dox 6 µM), (4) AntiSar/Zn/CS NPs/Dox-AuMNPs (Dox 6 µM), (5) Dox (0.25 µM), (6) Dox (0.5 µM), (7) Dox (1 µM). Data are expressed as an average of 12 repetitions. See to Materials and Methods Section for more information regarding experimental details.

Hemolytic activity in human erythrocytes as an alternative to in vivo testing was used as a potential screening method to evaluate CS NPs toxicity risk for intravenous administration. The impact of CS NPs on human red blood cells (RBCs) lysis was carried out spectrophotometrically recording the absorbance of hemoglobin at 570 nm. Each component of Zn/CS NPs have been investigated: CS, AuMNPs, Zn/CS NPs-AuMNPs and AntiSar/Zn/CS NPs-AuMNPs (see in Figure 8). As a positive control, the deionized water was used. From the results, it is obvious that the highest hemolytic activity shows unmodified CS (67%). Interestingly, after CS NPs formation, the hemolytic activity decreased by 30% (Figure 8A). This effect was similar in the case of Zn/CS NPs-AuMNPs and AntiSar/Zn/CS NPs-AuMNPs 40% respectively 41%. These results suggested rather that TPP addition the formation of zinc stabilized NPs leads to decrease CS toxicity and hemolytic activity. Our results are in good agreement with other studies [41,42,43]. Several experimental data suggested the coating of NPs by PEG or hyaluronic acid reduce hemolytic toxicity of NPs [44,45]. 

The yeast has been widely used as a test organism, since it is nonpathogenic, simple and easy to cultivate, has a fully annotated genome and shares a strong conservation at both metabolic and regulatory levels, with experimentally less accessible eukaryotes [46]. The viability of *S. cerevisiae* in the presence of main components of Zn/CS NPs (CS (2 mg/mL), AuMNPs (1 mg/mL), Zn/CS NPs-AuMNPs and AntiSar/Zn/CS NPs-AuMNPs (Dox 6 µM)) and Dox (0.25, 0.5 and 1 µM) was tested. The viability was determined as optical density at *λ* = 600 nm in time. From Figure 8B(a–e) is evident that the growth phase of *S. cerevisiae* is causing absorbance increase and on the contrary, *S. cerevisiae* death is reflected as optical density decrease (Figure 8B(a–e)). For the viability evaluation, the inhibitory effect of each compound was determined. Figure 8C shows, the highest inhibitory effect up to 15% with doxorubicin (0.25, 0.5 and 1 µM) in contrary to both designs of Zn/CS NPs (Zn/CS NPs-AuMNPs and AntiSar/Zn/CS NPs-AuMNPs) which showed the 3% inhibition effect. Surprisingly, CS alone and AuMNPs showed 6% and 16% inhibition effect, respectively. Toxicity results proved the encapsulation of Dox to the chitosan based nanotransporter had negligible effect on the *S. cerevisiae*. 

## 3. Discussion

The preparation of chitosan nanotransporters that can deliver the drug to the damaged tissues offers unquestionable benefits such as increasing the therapeutic window and reducing side effects. Many methods for the study of chitosan nanoparticles have been employed [10,47,48,49]. Based on the literature we applied ninhydrin assay for chitosan nanoparticle analysis [36]. For example, the ninhydrin assay has been successfully used for the study of CS NPs interaction with ligands such as glycyrrhizin and ferulic acid [50]. The decreased absorbance of Ruhemann’s purple indicated reduced amino group content of chitosan nanoparticles which could be considered as an additional evidence of the successful conjugation of selected ligands to the surface of chitosan nanoparticles [50]. Based on our results, the ninhydrin assay allows not only interaction determination but could give a reliable data about nanoparticle formation (see Figure 2). We supposed the relaxed structure of chitosan has an accessible amino group for ninhydrin reaction. While the TPP is added to the structure is strongly crosslinked and the nanoparticles are formed. The count of accessible amino groups decreases proportionally with the level of crosslinking and the structure of formed nanoparticles. This change was monitored by the decrease of the complex absorbance. Our results from ninhydrin assay determination of the CS NPs formation are comparable with Gan et al. who proved the influence of TPP addition and CS NPs formation using light scattering measurement [13]. In addition, our study was confirmed by independent electrochemical approach which enables us to monitor CS NPs formation indirectly. Signal of the free zinc ions is electrochemically detectable as reactions that deposit and dissolve metals [51,52].

[ML_n_]°_(solid)_ → e^−^ + [ML_n_]^+^_(solvated)_ stripping (1)

In the case of complex formation there is a shift in the observed signal but also a decrease in electrochemical response and changes in mechanism of charge transfer.

[ML_n_]°_(solid)_ /CS→ e^−^ + CS/[ML_n_]^+^_(solvated)_ stripping (2)

In comparison with literature, zinc influence of Dox entrapment efficiency has minimal effect and the encapsulation efficiency achieves similar level [53,54,55,56]. As Anitha et al. showed, the cargo release efficiency from chitosan nanocarrier is pH dependent [57] and after rapid doxorubicin elimination follows stable phase of the drug release [58]. The possibilities to modulate CS NPs for target therapy have been published by many authors. e.g., folate [59], aptamers [60], herceptin [61] were successfully bonded on the CS NPs surface. Antibody attachment and chitosan nanoparticle loading have been used by other authors with very promising results [62,63,64]. In summary, this mechanism for targeting cancer tissue is one of the most precise approaches to drug delivery nanosystems [26,65,66,67]. In addition, many studies showed low toxicity of chitosan polymer. Unfortunately, the evaluation of nanoparticle toxicity remains limited [68,69]. Our results confirmed the low toxicity of CS and also the low toxicity of doxorubicin loaded CS NPs compared to doxorubicin alone. *S. cerevisiae* as a broadly used model organism for anthracycline drug testing. Observations from other studies illustrate the higher resistance of *S. cerevisiae* against doxorubicin and the protective effect of glutathione and or metallothioneins [70]. The assessment of the results should be considered the phase of the cell cycle, which has a direct effect on the toxicity of doxorubicin [71,72,73,74]. Further investigations are necessary as well as a selective effect on healthy and prostate tumor tissue. 

The chitosan nanoparticles for doxorubicin delivery have been introduced by many authors [55]. Their popularity is given due to its good biocompatibility, possibilities for modifications, low cost and easy to prepare protocols. Based on several studies, zinc combined with chitosan has several applications such as: sensors [75], micronutrient nanocarrier [76] and nanocomposite coatings [77,78,79]. In our pilot experiment, zinc ions were incorporated into the CS NPs structure for a purpose of NP structure stabilization and passive targeting. The mechanism of stabilization could be explained by involving the amino groups of chitosan in the binding of zinc ions as well as ionic cross-linking with TPP molecules [76]. Although zinc is not used as a direct decoy for tumors in nanopharmaceuticals, several studies have pointed out the enhanced uptake of zinc ions in prostate cancer [80,81]. Normal prostate glands contain higher levels of zinc compared with cancerous tissues as well as the levels of MT. Generally, MT binds zinc more tightly than most other zinc proteins and constitutes a thermodynamic “sink” for zinc, in particular because the zinc concentration of MT is relatively high in comparison with other zinc proteins. Moreover, zinc could play therapeutic role in the cancer treatment. Jing et al. found that the zinc induces metallothionein overexpression which prevents cardiomyocytes from doxorubicin toxicity [82] and the apoptotic effect of zinc on malignant cells have been reviewed by Franklin et al. [83]. Additionally, MTs’ overexpression has been linked to chemoresistance in carcinoma cells [76]. These studies indicate the positive effect of zinc ions in the nanocarrier structure for doxorubicin delivery. Beside zinc, designed doxorubicin nanotransporter utilize other mechanism for target delivery of doxorubicin via attached antibodies. Generally, in conventional treatment the antibodies are oriented to specific receptors of malignant cell [84,85,86]. This research area has been explored by several promising studies and the findings are summarized in interesting reviews [87,88,89]. In fact, sarcosine is associated with glycine which is essential precursor for the synthesis of proteins, nucleic acids and lipids that are crucial to cancer cell growth [90]. Jain et al. showed that this metabolism of glycine could be used to target the rapidly proliferating cancer cells [91], possibly becoming a new anti-cancer treatment [92]. Since metabolites play an important role in tumor progression, it is necessary to study in detail. All the mechanisms, combination therapy approaches of cancer diseases and passive targeting to the product of cancer metabolism could improve the overall view of treatment of cancer diseases and gives a better chance for survival this serious disease.

## 4. Materials and Methods

### 4.1. Chemicals

Low molecular weight chitosan, Sodium Tripolyphosphate penta basic (TPP), DMSO, Sodium Acetate, Acetic acid, Doxorubicin HCl, Zinc Chloride, monoclonal Rabbit AntiChicken IgG and other chemical unless noted otherwise were purchased from Sigma-Aldrich (St. Louis, MO, USA). Ninhydrin, hydrindatin were purchased from (Ingos, Prague, Czech Republic). Polyclonal Chicken AntiSarcosine IgG was purchased from (Hena, Prague, Czech Republic). *S. cerevisiae* (ATCC 9763) were obtained from the Czech Collection of Microorganisms, Faculty of Science, Masaryk University, Brno, Czech Republic.

Aqueous solutions for size analysis were prepared using PURELAB^®^ Ultra (Elga, High Wycombe, UK) resistivity 18 MΩ-cm. For other purposes deionized water was used.

### 4.2. Chitosan Nanoparticles Preparation

In the case of Zn inner type of CS NPs, 12.5 mg of low MW chitosan was dissolved in 5 mL of 1% acetic acid. Doxorubicin (30 µM) and zinc (0.5 m/mL) was added to the chitosan solution under stirring, 30 rpm (3 h, 24 °C). After chitosan dissolution, the sodium tripolyphosphate (0.25% *w*/*v*) was added dropwise and incubated (30 min, 22 °C, 30 rpm) in rotator Multi-Rotator RS-60 BIOSAN. Zn outer type NPs were prepared in the same way, however the zinc (0.5 mg/mL) was added after TPP gelation. The mixture was stirred (30 rpm, 3 h, 24 °C).

### 4.3. AuNPs Preparation and Sarcosine/IgG Labeling

Gold nanoparticles (AuNPs) were prepared by mixing of 10 mL HAuCl_4_·3H_2_O solution (1 mM) and 250 µL C_6_H_5_Na_3_O_7_·2H_2_O (0.09 M). The mixture was stirred (30 rpm, 1 h in 20 °C) using magnetic stirrer (VMS-C4, VWR International s.r.o., Darmstadt, Germany). The light yellow solution turned to purple and the AuNPs were formed. 

Conjugation of AuNPs to sarcosine or IgG. 5 mL of AuNPs was added to 10 mL of 5 mM borate buffer (pH 8.8) under stirring (VMS-C4, VWR International s.r.o., Darmstadt, Germany). Subsequently, the 0.5 mL of Sar/IgG (1 mg/mL) was added dropwise. The mixture was incubated at 37 °C and at 350 rpm for 90 min (ThermoMixer F1.5, Eppendorf, Hamburg, Germany). Finally, the mixture was centrifuged (14,000 rpm, 22 °C and 10 min) and the formed pellet was diluted in 1 mL of 5 mM borate buffer. This step was repeated 4× times. After the last centrifugation, the pellet was dissolved in 2 mL of storage buffer (10 mM borate buffer (pH 8.8), 1% BSA). Prepared samples were stored in 4 °C. 

### 4.4. AuMNPs Preparation

1.5 g Fe(NO_3_)_3_·9H_2_O was dissolved in 80 mL water. 1.4 mL 25% NH_3_ was diluted in 8.6 mL water and 0.2 g NaBH_4_ was dissolved in this mixture. Mixture was stirred (30 rpm, 10 min at 22 °C) and after that the solution of Fe(NO_3_)_3_·9H_2_O was added. The color of solution turned to dark brown. The mixture was heated up to 100 °C for 2 h and stirred (30 rpm, overnight at 100 °C). The magnetic particles were separated from the solution via magnet and washed several times in water. The solution of HAuCl_4_ (25 mL, 1 mM) was added to the magnetic particles and stirred (30 rpm 1 h at 22 °C). Subsequently, the solution of C_6_H_5_Na_3_O_7_·2H_2_O (0.75 mL, 0.265 g/10 mL) was added to the mixture and stirred overnight. Finally, the gold magnetic nanoparticles (AuMNPs) were separated via magnet and dried (40 °C).

### 4.5. Ninyhdrin Assay for Chitosan Detection

The reaction solutions were then processed as described by [35,93]. The ninhydrin reagent was freshly prepared on the day of the assay by adding 25 mL of 4 M acetate buffer (pH 5.2) to 2 g ninhydrin and 0.3 g hydrindantin in 75 mL DMSO. For the assay, 75 µL of reagent was added to 100 µL of the sample in Eppendorf tube. The microtubes were immediately capped, briefly shaken by hand and heated by thermoblock for 30 min to allow the reaction to proceed. After cooling, 15 mL of a 50:50 ethanol:water mixture was added to each sample. The color intensity of the complex was spectrophotometrically evaluated as a measure of depolymerized chitosan activity. Accurately weighed depolymerized chitosan was dissolved in 1% *w*/*v* acetic acid. A blank solution was also prepared in an identical manner, wherein 1% *w*/*v* acetic acid was used instead of a chitosan solution to prepare the reaction mixture. The effect of the solvent system was mollified by calibrating the instrument to 100% transmittance of the blank solution. Relative absorbance for each CS NPs solution was calculated as follows:

RA = A of chitosan solution/A of standard solution(3)

### 4.6. Absorbance Measurements

Absorbance scan was carried out in the range from 300 to 700 nm by 2 nm steps. The samples for measurements (100 µL) were placed in 96 well UV plate (IAB, Prague, Czech Republic). All measurements were performed at 22 °C. (VWR, Darmstadt, Germany).

### 4.7. Fluorescence Reading

The fluorescence scan of doxorubicin was carried out using a fluorimeter (Infinite M200, TECAN, Männedorf, Switzerland). Measurement conditions were as follows: emission wavelength 510–700 nm, excitation wavelength 480 nm, gain 200, number of flashes 35, integrated time 50 µs. The samples for measurements (100 µL) were placed in 96 well UV plate (IAB, Prague, Czech Republic).

### 4.8. Electrochemical Determination of Zinc Ions

Determination of Zn^2+^ by difference pulse voltammetry (DPV) was performed at 663 VA Stand (Metrohm, Zofingen, Switzerland) connected with Analyzer (Metrohm). A hanging mercury drop electrode (HMDE) with a drop area of 0.4 mm^2^ was employed as the working electrode. An Ag/AgCl/3 M KCl electrode acted as the reference and platinum electrode was auxiliary. The analyzed samples were deoxygenated prior to measurements by purging with argon (99.999%). Acetate buffer (0.2 M sodium acetate and 0.2 acetic acid, pH = 5) was used as a supporting electrolyte. The parameters of the measurement were as follows: initial potential 0 V, end potential −1.7 V, deoxygenating with argon 120 s, accumulation time 120 s, step potential 5 mV, modulation amplitude 25 mV, volume of injected sample: 50 µL, volume of measurement cell 10 mL. 

### 4.9. CS NPs Magnetic Separation

The CS NPs were prepared according to Section 4.6. 12.5 mg of low MW chitosan was dissolved in 5 mL of 1% acetic acid. Doxorubicin (30 µM) and zinc (0.5 mg/mL) and AuMNPs (1 mg/mL) were added to the chitosan solution under stirring (3 h, 24 °C). After chitosan dissolution, the sodium tripolyphosphate (0.25% *w*/*v*) was added dropwise and incubated in rotator Multi-Rotator RS-60 BIOSAN. Magnetic AntiSar/Zn/CS NPs/Dox-AuMNPs was immobilized via magnet and washed using PBS buffer prior to each analysis. 

### 4.10. CS NPs-Dox Entrapment Efficiency

The entrapment efficiency (EE) of the CS NPs was expressed as the actual doxorubicin loading of the NPs referred to the initial amount of doxorubicin used. The encapsulated Dox concentration was calculated from the calibration curve of fluorescence signal. EE (%) was calculated using the following equation:

EE (%) = amount of Dox in NPs/amount of initial Dox used × 100(4)

### 4.11. Cumulative Release of Dox from CS NPs

100 µL of CS NPs/Dox-AuMNPs were added to 4 (1.5 mL) tubes (Eppendorf, Hamburg, Germany). Into each tube 1–4 was added 500 µL of 1 mM phosphate buffer pH 5, 6 and 8 or whole blood. Samples were incubated on a rotator (Multi RS-60, Biosan, Latvia) for 30 min, 30 rpm 22 °C. Subsequently, the NPs were immobilized via magnet and washed with 500 µL of 1% acetic acid. Finally, CSNPs/Dox-AuMNPs was re-suspended in 300 µL of 1% acetic acid and transferred to the UV plate (IAB, Prague, Czech Republic). The process was repeated until the end time of the analysis. 

### 4.12. Dot Blot Assay

On the nitrocelulose membrane 0.45 µm pore diameter (SERVA, Heidelberg, Germany) was pipetted 1–2 µL of Rabbit AntiChicken antibody (30 mg/mL). After drying, the membrane was blocked by 1% BSA in PBS buffer. After 30 min incubation in the rotator the membrane was soaked in the CSNPs (AntiSar/CS NPS/Dox-AuMNPs) solution for 1 h. The incubation was carried out in the 50 mL tube on rotator (15 rpm). The membrane was 3× washed using PBS-T. Further, the membrane was soaked in AuNPs labeled antibody (40 mg/mL) or sarcosine (1 mg/mL) for 1 h. Each sample was washed from unbounded parts by PBS-T buffer. After dying the membrane, the samples were prepared for evaluation.

### 4.13. ELISA

AntiSarCS NPs/Dox immunodetection was tested by ELISA. Microtitration plate was coated with 100 µL per well of primary (AntiChicken) antibody diluted 1:1000 in 0.05 M carbonate buffer (32 mM Na_2_CO_3_ and 0.068 M NaHCO_3_, pH 9.6) at 37 °C for 1 h. After coating the free surface the wells were blocked with 150 µL per well of 1% BSA (*w*/*v*) in PBS (137 mM NaCl, 2.7 mM KCl, 1.4 mM NaH_2_PO_4_ and 4.3 mM Na_2_HPO_4_, pH 7.4) for 30 min at 37 °C, then the wells were washed 5× with 350 µL of 0.05% (*v*/*v*) PBS-T (Hydroflex, TECAN, Männedorf, Switzerland). Then, 100 µL of the sample of AntiSar/CS NPs/Dox-AuMNPs were added and the microplate was incubated at 37 °C for 1 h. After washing with PBS-T, 100 µL of secondary (AntiChicken) antibody AuNPs conjugated in dilution 1:100 in PBS was added and the plate was incubated for 60 min at 37 °C. After incubation and washing 100 µL of 0.45 mM TMB in 0.5 M acetate buffer adjusted to pH 4 with citric acid with 2.9% (*v*/*v*) of H_2_O_2_ was added. The absorbance was read at 650 nm during 45 min with 5 min steps (Infinite M200 Pro, TECAN, Männedorf, Switzerland).

### 4.14. Hemolytic Assay

Hemolytic assay was carried out on human erythrocytes. Plasma from the fresh blood sample collected from a laboratory staff (RBC 5.16 × 10^12^ L; HBG 161 g/L; MCV 83.6 fl Hematology analyzer Mindray BC-5500, Shenzhen, China) was removed by multiple washings with 150 mM sodium chloride and centrifuged at 5000 rpm for 5 min. Then, prepared samples were mixed with the RBC and incubated for 1 h at 37 °C. PBS and 0.1% 18 MΩ water was used as negative and positive control. After completion of the incubation period, the cells were centrifuged and the absorbance of the supernatant containing lysed erythrocytes was measured at 540 nm. The percentage of hemolysis was determined by the following equation:

(5) $$\%\;\mathrm{Hemolysis}=\;\left[\frac{A_{t}-A_{c}}{A_{100\%}-A_{c}}\right]\times 100$$

where A_t_ is the absorbance of the supernatant from samples incubated with the particles, A_c_ is the absorbance of the supernatant from the negative control (PBS) and A_100%_ is the absorbance of the positive control supernatant. 

### 4.15. Inhibition Assay

For the determination of growth curves the LB medium (trypton, yeast extract, NaCl) was used. Cultivated microorganisms (*S. scerevisiae*) were diluted using LB media to the absorption 0.1 at 600 nm before measurement. To each well was pipetted 250 µL of diluted *S. cerevisiae* and 50 µL of the tested sample was added. As a control, the 300 µL of LB media was added to the separate well. The measurement was carried out on the Tecan infinite 200 Pro Multifunctional Reader (TECAN, Männedorf, Switzerland) at 600 nm for 18 h and 37 °C. The absorbance value was recorded after 30 min. Each sample was analyzed in five independent repetitions. The results were expressed as an average value. 

### 4.16. Data Treatment and Descriptive Statistics

The experimental work was carried out in using the three independent experiments. The analysis of each sample was carried out 5 times. Obtained data are presented as an average value. No experimental subjects were excluded from the proposed experimental studies. All the obtained data are stored in the Ladys database. If possible, data was processed and evaluated mathematically and statistically in the Ladys database. Photographs were processed using the ColorTest program, which assigns intensity to the individual pixels of the studied image in the color area. 

## 5. Conclusions

A detailed interaction study of chitosan nanotransporter, zinc ions and TPP has been performed. The Zn^2+^ ion concentration shows the strong effect on crosslinking and coiling of CS NPs with the result of better nanoparticle stabilization confirmed by ninhydrin assay and electrochemical determination. The entrapment efficiency for doxorubicin of designed CS NPs was 20%. The highest drug release from the CS nanocarrier appears after 120 min in the pH 5, 6, 8 and blood. Functionality and ability of the AntiSar/CS NPs/Dox to bind on the sarcosine molecule was proved using dot blot analysis and ELISA. Observed hemolytic activity of the nanotransporter was 40% and the 20% inhibition activity on the experimental model of *S. cerevisiae*. Zn/CS nanotransporter for doxorubicin delivery showed good stability, doxorubicin binding capacity and low toxicity. In this study, we have demonstrated a design of nanotransporter suitable for doxorubicin. Due to the independent analytical methods, the functionality and non-toxicity have been confirmed. Our results indicate the suitability of nanocarrier for testing in vitro on cancerous tissue cultures.

## Figures and Tables

**Table 1:** Analytical parameters of ninhydrin assay for chitosan determination.

Substance	Regression Equation	Linear Dynamic Range (µg/mL)	R^2^	LOD (µg/mL)	LOQ (µg/mL)	RSD * (%)
Chitosan A_408_	*y* = 1.7469*x* + 0.1037	8–500	0.9965	2	17	2
Chitosan A_572_	*y* = 1.5443*x* + 0.0384	8–500	0.9969	2	19	2

* *n* = 5.

## Abbreviations

AntiSar	Anti-sarcosine Chicken Antibodies
AuMNPs	Gold Magnetic Nanoparticles
AuNPs	Gold Nanoparticles
AuSar	AuNPs Labeled Sarcosine
BSA	Bovine Serum Albumin
CS	Chitosan
MT	Metallothionein
NPs	Nanoparticles
PC	Prostate Cancer
RBCs	Red Blood Cells
TPP	Sodium Tripolyphosphate

## References

[1] Lal N., Dubey J., Gaur P., Verma N., Verma A. (2017). Chitosan based in situ forming polyelectrolyte complexes: A potential sustained drug delivery polymeric carrier for high dose drugs. Mater. Sci. Eng. C Mater. Biol. Appl..

[2] Sen V.D., Sokolova E.M., Neshev N.I., Kulikov A.V., Pliss E.M. (2017). Low molecular chitosan-(poly)nitroxides: Synthesis and evaluation as antioxidants on free radical-induced erythrocyte hemolysis. React. Funct. Polym..

[3] Niaz T., Nasir H., Shabbir S., Rehman A., Imran M. (2016). Polyionic hybrid nano-engineered systems comprising alginate and chitosan for antihypertensive therapeutics. Int. J. Biol. Macromol..

[4] Ellis C.E., Korbutt G.S., Jennings J.A., Bumgardner J.D. (2017). Chitosan-based biomaterials for treatment of diabetes. Chitosan Based Biomaterials; Volume 2; Tissue Engineering and Therapeutics.

[5] Pangestuti R., Kim S.-K. (2010). Neuroprotective properties of chitosan and its derivatives. Mar. Drugs.

[6] Shariatinia Z., Fazli M. (2015). Mechanical properties and antibacterial activities of novel nanobiocomposite films of chitosan and starch. Food Hydrocoll..

[7] Behera S.S., Das U., Kumar A., Bissoyi A., Singh A.K. (2017). Chitosan/TiO_2_ composite membrane improves proliferation and survival of l929 fibroblast cells: Application in wound dressing and skin regeneration. Int. J. Biol. Macromol..

[8] Park K. (2017). Chitosan-gelatin-platelet gel composite scaffold for bone regeneration. J. Controll. Release.

[9] Xu H., Matysiak S. (2017). Effect of ph on chitosan hydrogel polymer network structure. Chem. Commun..

[10] Desai K.G.H. (2016). Chitosan nanoparticles prepared by ionotropic gelation: An overview of recent advances. Crit. Rev. Ther. Drug Carr. Syst..

[11] Huang Y., Lapitsky Y. (2017). On the kinetics of chitosan/tripolyphosphate micro- and nanogel aggregation and their effects on particle polydispersity. J. Colloid Interface Sci..

[12] Sacco P., Paoletti S., Cok M., Asaro F., Abrami M., Grassi M., Donati I. (2016). Insight into the ionotropic gelation of chitosan using tripolyphosphate and pyrophosphate as cross-linkers. Int. J. Biol. Macromol..

[13] Gan Q., Wang T., Cochrane C., McCarron P. (2005). Modulation of surface charge, particle size and morphological properties of chitosan-tpp nanoparticles intended for gene delivery. Colloids Surf. B Biointerfaces.

[14] Yata V.K., Ghosh S.S. (2012). Investigating structure and fluorescence properties of green fluorescent protein released from chitosan nanoparticles. Mater. Lett..

[15] Shanmugam R., Priyanka D.L., Madhuri K., Gowthamarajan K., Karri V., Kumar C.K.A., Murali P. (2017). Formulation and characterization of chitosan encapsulated phytoconstituents of curcumin and rutin nanoparticles. Int. J. Biol. Macromol..

[16] Duarte A.P., Tavares E.J.M., Alves T.V.G., de Moura M.R., da Costa C.E.F., Silva J.O.C., Costa R.M.R. (2017). Chitosan nanoparticles as a modified diclofenac drug release system. J. Nanopart. Res..

[17] Mendelovits A., Prat T., Gonen Y., Rytwo G. (2012). Improved colorimetric determination of chitosan concentrations by dye binding. Appl. Spectrosc..

[18] Badawy M.E.I. (2012). A new rapid and sensitive spectrophotometric method for determination of a biopolymer chitosan. Int. J. Carbohydr. Chem..

[19] Assa F., Jafarizadeh-Malmiri H., Ajamein H., Vaghari H., Anarjan N., Ahmadi O., Berenjian A. (2017). Chitosan magnetic nanoparticles for drug delivery systems. Crit. Rev. Biotechnol..

[20] Babu A., Ramesh R. (2017). Multifaceted applications of chitosan in cancer drug delivery and therapy. Mar. Drugs.

[21] Hong S.-C., Yoo S.-Y., Kim H., Lee J. (2017). Chitosan-based multifunctional platforms for local delivery of therapeutics. Mar. Drugs.

[22] Duttagupta D.S., Jadhav V.M., Kadam V.J. (2015). Chitosan: A propitious biopolymer for drug delivery. Curr. Drug Deliv..

[23] Landriscina A., Rosen J., Friedman A.J. (2015). Biodegradable chitosan nanoparticles in drug delivery for infectious disease. Nanomedicine.

[24] Sarvaiya J., Agrawal Y.K. (2015). Chitosan as a suitable nanocarrier material for anti-alzheimer drug delivery. Int. J. Biol. Macromol..

[25] Lee J., Yun K.S., Choi C.S., Shin S.H., Ban H.S., Rhim T., Lee S.K., Lee K.Y. (2012). T cell-specific sirna delivery using antibody-conjugated chitosan nanoparticles. Bioconjug. Chem..

[26] Sau S., Alsaab H.O., Kashaw S.K., Tatiparti K., Iyer A.K. (2017). Advances in antibody-drug conjugates: A new era of targeted cancer therapy. Drug Discov. Today.

[27] Sreekumar A., Poisson L.M., Rajendiran T.M., Khan A.P., Cao Q., Yu J., Laxman B., Mehra R., Lonigro R.J., Li Y. (2009). Metabolomic profiles delineate potential role for sarcosine in prostate cancer progression. Nature.

[28] Kim H.M., Lee Y.K., Koo J.S. (2016). Expression of sarcosine-metabolizing enzymes in thyroid cancer. Int. J. Clin. Exp. Pathol..

[29] Cha Y.J., Kim D.H., Jung W.H., Koo J.S. (2014). Expression of sarcosine metabolism-related proteins according to metastatic site in breast cancer. Int. J. Clin. Exp. Pathol..

[30] Robinson D.R., Wu Y.M., Lonigro R.J., Vats P., Cobain E., Everett J., Cao X.H., Rabban E., Kumar-Sinha C., Raymond V. (2017). Integrative clinical genomics of metastatic cancer. Nature.

[31] Bizon A., Jedryczko K., Milnerowicz H. (2017). The role of metallothionein in oncogenesis and cancer treatment. Postepy Hig. Med. Dosw..

[32] Krizkova S., Ryvolova M., Hrabeta J., Adam V., Stiborova M., Eckschlager T., Kizek R. (2012). Metallothioneins and zinc in cancer diagnosis and therapy. Drug Metab. Rev..

[33] Costello L.C., Franklin R.B. (2016). A comprehensive review of the role of zinc in normal prostate function and metabolism; and its implications in prostate cancer. Arch. Biochem. Biophys..

[34] Sheng S.J., Kraft J.J., Schuster S.M. (1993). A specific quantitative colorimetric assay for l-asparagine. Anal. Biochem..

[35] Prochazkova S., Vårum K.M., Ostgaard K. (1999). Quantitative determination of chitosans by ninhydrin. Carbohydr. Polym..

[36] Leane M.M., Nankervis R., Smith A., Illum L. (2004). Use of the ninhydrin assay to measure the release of chitosan from oral solid dosage forms. Int. J. Pharm..

[37] Raja M.A., Arif M., Feng C., Zeenat S., Liu C.G. (2017). Synthesis and evaluation of ph-sensitive, self-assembled chitosan-based nanoparticles as efficient doxorubicin carriers. J. Biomater. Appl..

[38] Lim E.K., Huh Y.M., Yang J., Lee K., Suh J.S., Haam S. (2011). Ph-triggered drug-releasing magnetic nanoparticles for cancer therapy guided by molecular imaging by mri. Adv. Mater..

[39] Bekale L., Agudelo D., Tajmir-Riahi H.A. (2015). Effect of polymer molecular weight on chitosan-protein interaction. Colloids Surf. B Biointerfaces.

[40] Docekalova M., Uhlirova D., Stankova M., Kepinska M., Sochor J., Milnerowicz H., Babula P., Fernandez C., Brazdova M., Zidkova J. (2017). Characterisation of peroxidase-like activity of thermally synthesized gold nanoparticles. Proceedings of the Nanocon 2016, 8th International Conference on Nanomaterials.

[41] Wang Y.B., Zhou J.R., Liu L., Huang C.J., Zhou D.Q., Fu L.L. (2016). Characterization and toxicology evaluation of chitosan nanoparticles on the embryonic development of zebrafish, danio rerio. Carbohydr. Polym..

[42] Hu Y.L., Qi W., Han F., Shao J.Z., Gao J.Q. (2011). Toxicity evaluation of biodegradable chitosan nanoparticles using a zebrafish embryo model. Int. J. Nanomed..

[43] Choi Y.J., Gurunathan S., Kim D., Jang H.S., Park W.J., Cho S.G., Park C., Song H., Seo H.G., Kim J.H. (2016). Rapamycin ameliorates chitosan nanoparticle-induced developmental defects of preimplantation embryos in mice. Oncotarget.

[44] De Salamanca A.E., Diebold Y., Calonge M., Garcia-Vazquez C., Callejo S., Vila A., Alonso M.J. (2006). Chitosan nanoparticles as a potential drug delivery system for the ocular surface: Toxicity, uptake mechanism and in vivo tolerance. Investig. Ophthalmol. Vis. Sci..

[45] Xu Y.R., Asghar S., Yang L., Chen Z.P., Li H.Y., Shi W.W., Li Y.B., Shi Q.Q., Ping Q.N., Xiao Y.Y. (2017). Nanoparticles based on chitosan hydrochloride/hyaluronic acid/peg containing curcumin: In Vitro evaluation and pharmacokinetics in rats. Int. J. Biol. Macromol..

[46] Liu W., Li L., Ye H., Chen H., Shen W., Zhong Y., Tian T., He H. (2017). From saccharomyces cerevisiae to human: The important gene co-expression modules. Biomed. Rep..

[47] Key J., Park K. (2017). Multicomponent, tumor-homing chitosan nanoparticles for cancer imaging and therapy. Int. J. Mol. Sci..

[48] Fu S., Xia J., Wu J. (2016). Functional chitosan nanoparticles in cancer treatment. J. Biomed. Nanotechnol..

[49] Bugnicourt L., Ladaviere C. (2016). Interests of chitosan nanoparticles conically cross-linked with tripolyphosphate for biomedical applications. Prog. Polym. Sci..

[50] El-Marakby E.M., Hathout R.M., Taha I., Mansour S., Mortada N.D. (2017). A novel serum-stable liver targeted cytotoxic system using valerate-conjugated chitosan nanoparticles surface decorated with glycyrrhizin. Int. J. Pharm..

[51] Yu J.X., Wang L., Su L., Ai X.P., Yang H.X. (2003). Temperature effects on the electrodeposition of zinc. J. Electrochem. Soc..

[52] Kudr J., Hoai Viet N., Gumulec J., Nejdl L., Blazkova I., Ruttkay-Nedecky B., Hynek D., Kynicky J., Adam V., Kizek R. (2015). Simultaneous automatic electrochemical detection of zinc, cadmium, copper and lead ions in environmental samples using a thin-film mercury electrode and an artificial neural network. Sensors.

[53] Di Martino A., Sedlarik V. (2014). Amphiphilic chitosan-grafted-functionalized polylactic acid based nanoparticles as a delivery system for doxorubicin and temozolomide co-therapy. Int. J. Pharm..

[54] Xiong W., Li L., Wang Y., Yu Y., Wang S., Gao Y., Liang Y., Zhang G., Pan W., Yang X. (2016). Design and evaluation of a novel potential carrier for a hydrophilic antitumor drug: Auricularia auricular polysaccharide-chitosan nanoparticles as a delivery system for doxorubicin hydrochloride. Int. J. Pharm..

[55] Janes K.A., Fresneau M.P., Marazuela A., Fabra A., Alonso M.J. (2001). Chitosan nanoparticles as delivery systems for doxorubicin. J. Controll. Release.

[56] Soares P.I.P., Sousa A.I., Silva J.C., Ferreira I.M.M., Novo C.M.M., Borges J.P. (2016). Chitosan-based nanoparticles as drug delivery systems for doxorubicin: Optimization and modelling. Carbohydr. Polym..

[57] Anitha A., Deepagan V.G., Rani V.V.D., Menon D., Nair S.V., Jayakumar R. (2011). Preparation, characterization, in vitro drug release and biological studies of curcumin loaded dextran sulphate-chitosan nanoparticles. Carbohydr. Polym..

[58] Esfandiarpour-Boroujeni S., Bagheri-Khoulenjani S., Mirzadeh H., Amanpour S. (2017). Fabrication and study of curcumin loaded nanoparticles based on folate-chitosan for breast cancer therapy application. Carbohydr. Polym..

[59] Yang C.L., Chen J.P., Wei K.C., Chen J.Y., Huang C.W., Liao Z.X. (2017). Release of doxorubicin by a folate-grafted, chitosan-coated magnetic nanoparticle. Nanomaterials.

[60] Chen X., Zhu X.Y., Li L., Xian G.J., Wang W., Ma D.W., Xie L. (2013). Investigation on novel chitosan nanoparticle-aptamer complexes targeting tgf-beta receptor II. Int. J. Pharm..

[61] Arya G., Vandana M., Acharya S., Sahoo S.K. (2011). Enhanced antiproliferative activity of herceptin (HER2)-conjugated gemcitabine-loaded chitosan nanoparticle in pancreatic cancer therapy. Nanomed. Nanotechnol. Biol. Med..

[62] Zhu R., Zhang C.-G., Liu Y., Yuan Z.-Q., Chen W.-L., Yang S.-D., Li J.-Z., Zhu W.-J., Zhou X.-F., You B.-G. (2015). Cd147 monoclonal antibody mediated by chitosan nanoparticles loaded with α-hederin enhances antineoplastic activity and cellular uptake in liver cancer cells. Sci. Rep..

[63] Yousefpour P., Atyabi F., Vasheghani-Farahani E., Movahedi A.-A.M., Dinarvand R. (2011). Targeted delivery of doxorubicin-utilizing chitosan nanoparticles surface-functionalized with anti-her2 trastuzumab. Int. J. Nanomed..

[64] Zhao L., Yang G., Shi Y., Su C., Chang J. (2015). Co-delivery of gefitinib and chloroquine by chitosan nanoparticles for overcoming the drug acquired resistance. J. Nanobiotechnol..

[65] Shargh V.H., Hondermarck H., Liang M. (2016). Antibody-targeted biodegradable nanoparticles for cancer therapy. Nanomedicine.

[66] Goodall S., Jones M.L., Mahler S. (2015). Monoclonal antibody-targeted polymeric nanoparticles for cancer therapy-future prospects. J. Chem. Technol. Biotechnol..

[67] Zhu Y., Choi S.H., Shah K. (2015). Multifunctional receptor-targeting antibodies for cancer therapy. Lancet Oncol..

[68] Svirshchevskaya E.V., Zubareva A.A., Boyko A.A., Shustova O.A., Grechikhina M.V., Shagdarova B.T., Varlamov V.P. (2016). Analysis of toxicity and biocompatibility of chitosan derivatives with different physico-chemical properties. Appl. Biochem. Microbiol..

[69] Zubareva A., Shagdarova B., Varlamov V., Kashirina E., Svirshchevskaya E. (2017). Penetration and toxicity of chitosan and its derivatives. Eur. Polym. J..

[70] Saenko Y.V., Shutov A.M., Rastorgueva E.V. (2010). Doxorubicin and menadione decrease cell proliferation of saccharomyces cerevisiae by different mechanisms. Cell Tissue Biol..

[71] Nguyen T.T.T., Lim Y.J., Fan M.H.M., Jackson R.A., Lim K.K., Ang W.H., Ban K.H.K., Chen E.S. (2016). Calcium modulation of doxorubicin cytotoxicity in yeast and human cells. Genes Cells.

[72] Westmoreland T.J., Wickramasekara S.M., Guo A.Y., Selim A.L., Winsor T.S., Greenleaf A.L., Blackwell K.L., Olson J.A., Marks J.R., Bennett C.B. (2009). Comparative genome-wide screening identifies a conserved doxorubicin repair network that is diploid specific in saccharomyces cerevisiae. PLoS ONE.

[73] Demir A.B., Koc A. (2015). High-copy overexpression screening reveals pdr5 as the main doxorubicin resistance gene in yeast. PLoS ONE.

[74] Xia L., Jaafar L., Cashikar A., Flores-Rozas H. (2007). Identification of genes required for protection from doxorubicin by a genome-wide screen in saccharomyces cerevisiae. Cancer Res..

[75] Hooda V., Archita (2018). Enzymes loaded chitosan/coconut fibre/zinc oxide nanoparticles strip for polyamine determination. Food Chem..

[76] Deshpande P., Dapkekar A., Oak M.D., Paknikar K.M., Rajwade J.M. (2017). Zinc complexed chitosan/tpp nanoparticles: A promising micronutrient nanocarrier suited for foliar application. Carbohydr. Polym..

[77] Al-Naamani L., Dobretsov S., Dutta J., Burgess J.G. (2017). Chitosan-zinc oxide nanocomposite coatings for the prevention of marine biofouling. Chemosphere.

[78] Noshirvani N., Ghanbarzadeh B., Mokarram R.R., Hashemi M., Coma V. (2017). Preparation and characterization of active emulsified films based on chitosan-carboxymethyl cellulose containing zinc oxide nano particles. Int. J. Biol. Macromol..

[79] Wang H.J., Liu S.L., Zhang A.K., Li K.W., Oderinde O., Yao F., Fu G.D. (2017). Zinc ion-induced formation of hierarchical N-succinyl chitosan film. J. Appl. Polym. Sci..

[80] Costello L.C., Franklin R.B., Zou J., Feng P., Bok R., Swanson M.G., Kurhanewicz J. (2011). Human prostate cancer zip1/zinc/citrate genetic/metabolic relationship in the tramp prostate cancer animal model. Cancer Biol. Ther..

[81] Dambal S., Baumann B., McCray T., Williams L., Richards Z., Deaton R., Prins G.S., Nonn L. (2017). The mir-183 family cluster alters zinc homeostasis in benign prostate cells, organoids and prostate cancer xenografts. Sci. Rep..

[82] Jing L., Li L.Z., Zhao J., Sun Z.W., Peng S.Q. (2016). Zinc-induced metallothionein overexpression prevents doxorubicin toxicity in cardiomyocytes by regulating the peroxiredoxins. Xenobiotica.

[83] Franklin R.B., Costello L.C. (2009). The important role of the apoptotic effects of zinc in the development of cancers. J. Cell. Biochem..

[84] Pang S.-T., Lin F.-W., Chuang C.-K., Yang H.-W. (2017). Co-delivery of docetaxel and p44/42 mapk sirna using PSMA antibody-conjugated BSA-PEI layer-by-layer nanoparticles for prostate cancer target therapy. Macromol. Biosci..

[85] Daniels-Wells T.R., Helguera G., Leuchter R.K., Quintero R., Kozman M., Rodriguez J.A., Ortiz-Sanchez E., Martinez-Maza O., Schultes B.C., Nicodemus C.F. (2013). A novel ige antibody targeting the prostate-specific antigen as a potential prostate cancer therapy. BMC Cancer.

[86] Nagesh P.K.B., Johnson N., Boya V.K.N., Chowdhury P., Ganju A., Hafeez B., Khan S., Jaggi M., Chauhan S.C., Yallapu M.M. (2016). PSMA antibody functionalized docetaxel-loaded magnetic nanoparticles for prostate cancer therapy. Cancer Res..

[87] Lukey M.J., Katt W.P., Cerione R.A. (2017). Targeting amino acid metabolism for cancer therapy. Drug Discov. Today.

[88] Roy D., Sheng G.Y., Herve S., Carvalho E., Mahanty A., Yuan S.T., Sun L. (2017). Interplay between cancer cell cycle and metabolism: Challenges, targets and therapeutic opportunities. Biomed. Pharmacother..

[89] Sidaway P. (2017). Prostate cancer: Targeting lipid metabolism. Nat. Rev. Urol..

[90] Amelio I., Cutruzzola F., Antonov A., Agostini M., Melino G. (2014). Serine and glycine metabolism in cancer. Trends Biochem. Sci..

[91] Jain M., Nilsson R., Sharma S., Madhusudhan N., Kitami T., Souza A.L., Kafri R., Kirschner M.W., Clish C.B., Mootha V.K. (2012). Metabolite profiling identifies a key role for glycine in rapid cancer cell proliferation. Science.

[92] Heger Z., Polanska H., Rodrigo M.A.M., Guran R., Kulich P., Kopel P., Masarik M., Eckschlager T., Stiborova M., Kizek R. (2016). Prostate tumor attenuation in the nu/nu murine model due to anti-sarcosine antibodies in folate-targeted liposomes. Sci. Rep..

[93] Sabnis S., Block L.H. (2000). Chitosan as an enabling excipient for drug delivery systems. I. Molecular modifications. Int. J. Biol. Macromol..

